# Research Progress on Chloride Channel- and Transporter- Related Gene Families in Plants

**DOI:** 10.3390/ijms27146371

**Published:** 2026-07-17

**Authors:** Yiru Song, Chen Meng, Syeda Wajeeha Gillani, Meng Wang, Xueli Lu, Yiqiang Li, Zongchang Xu

**Affiliations:** 1 College of Agronomy, Qingdao Agricultural University, Qingdao 266109, China; 2 Marine Agriculture Research Center, Tobacco Research Institute of Chinese Academy of Agricultural Sciences, Qingdao Key Laboratory of Coastal Saline-Alkali Land Resources Mining and Biological Breeding, Qingdao 266100, China

**Keywords:** plant chloride channels, abiotic stress response, regulation mechanism, plant nutrition, crop improvement, salt stress

## Abstract

Chloride (Cl^−^) is an essential micronutrient for plants that supports multiple physiological functions throughout plant growth and development. Its effects are strongly concentration-dependent: low Cl^−^ availability promotes beneficial physiological processes, whereas excessive accumulation can induce cytotoxicity. In plants, the movement of Cl^−^ across plasma and organellar membranes is primarily mediated by three principal channel and transporter families: chloride channels (CLC), aluminum-activated malate transporters (ALMT), and slow anion channel-associated homologs (SLAC/SLAH). These families differ in gating mechanisms, ion selectivity, transport properties, and subcellular localization. This review synthesizes current knowledge of plant chloride transport proteins, with emphasis on their phylogenetic distribution, structural organization, and functional diversification. We summarize their core physiological roles in stomatal regulation, water-use efficiency, nutrient uptake, ion homeostasis, growth modulation, and abiotic stress tolerance. We also discuss how their activities are regulated by post-translational modifications, notably phosphorylation and dephosphorylation, as well as by ion concentrations, pH shifts, and phytohormone signaling. Unlike earlier reviews that primarily focused on individual transporter families or specific stress responses, this work provides an integrated framework linking structure–function relationships with regulatory networks. It also evaluates recent advances in high-resolution structural biology, electrophysiological approaches, and in vivo imaging techniques. Furthermore, we delineate current technical bottlenecks and unresolved questions, such as the molecular determinants of substrate specificity and potential cross-talk among transporter families, and propose future directions for crop improvement. By integrating structural, physiological, and regulatory perspectives, this review aims to serve as a valuable reference and stimulate interdisciplinary research on plant chloride biology.

## 1. Introduction

Chlorine, an essential micronutrient for plant growth and development, primarily exists as chloride ions (Cl^−^) in soils and plants. Naturally occurring available soil Cl^−^ generally meets the nutritional requirements of crops [1]. Chlorine is also present in certain chlorinated phytohormone-related compounds, such as 4-chloroindole-3-acetic acid, which has been associated with growth-promoting effects in plants [2]. Chloride contributes to several essential physiological processes, including cellular osmotic regulation, stomatal movement, photosynthesis, disease resistance, stress tolerance, and normal plant growth [3]. Although Cl^−^ is required only in small amounts, excessive accumulation can disrupt cellular homeostasis and cause toxicity [1]. Leaf scorch is a typical symptom of Cl^−^ toxicity, and severe accumulation can inhibit growth and reduce plant yield [1]. Based on critical Cl^−^ tolerance thresholds, crops can be classified into three categories: highly tolerant (>600 mg/kg), moderately tolerant (300–600 mg/kg), and low tolerant (150–300 mg/kg). The moderately tolerant group can be further subdivided into two subcategories: moderately high-tolerant (450–600 mg/kg) and moderately low-tolerant (300–450 mg/kg) [4].

Because Cl^−^ participates in numerous cellular and physiological processes, its uptake, intracellular distribution, and compartmentalization depend on membrane-localized channels and transport proteins. Recent studies have shown that Cl^−^ channels and transporters are distributed across multiple plant membranes, including the plasma membrane, vacuolar membrane, endoplasmic reticulum membrane, mitochondrial membrane [3], chloroplast envelope [5], and thylakoid membrane [6]. These proteins contribute to cell turgor maintenance, osmotic regulation, ion homeostasis, intracellular pH regulation, stomatal movement, nutrient transport, metal tolerance, and signal transduction [7]. The major gene families associated with Cl^−^ transport in plants include the chloride channel/transporter (CLC) family. Other protein families, such as aluminum-activated malate transporter (ALMT), slow anion channel-associated homolog (SLAC/SLAH), nitrate transporter 1/Peptide transporter family proteins (NRT1/NPF), cation-chloride cotransporters (CCCs), and multidrug and toxin extrusion proteins (MATEs), also contribute to Cl^−^ transport, ion distribution, and stress responses [8].

This review summarizes the discovery and development of research on plant chloride channels and transporters. It further describes the structural characteristics of major Cl^−^-associated gene families, discusses their physiological functions and regulatory mechanisms, and reviews recent technological advances and potential agricultural applications. It also outlines current challenges and future research outlooks, providing a useful reference for further research on chloride transport-mediated regulation in plants.

## 2. Discovery History of Chloride Channels and Transporters

Before the 1980s, research on chloride channels was mainly limited to physiological observations. Although specific molecular entities had not yet been identified, plant physiological studies suggested the existence of membrane systems involved in Cl^−^ transport. Subsequently, the first chloride channel gene (named CLC-0) was isolated from *Torpedo marmorata* using *Xenopus* oocyte expression technology [9]. This discovery provided an important molecular reference for the subsequent identification of homologous chloride channel genes in plants.

With the development of molecular cloning technology, research on plant chloride channels made substantial progress. The first plant CLC family member (CLC-NT1) was cloned from tobacco cDNA and shown to encode a 780-amino-acid protein with multiple transmembrane domains. This represented the first clearly identified chloride channel-related gene in plants [10]. Around the same period, four CLC members (AtCLC-a, AtCLC-b, AtCLC-c, and AtCLC-d) were first reported in *Arabidopsis thaliana*, establishing a foundation for systematic investigation of the plant CLC family. Subsequently, three additional members, AtCLC-e, AtCLC-f, and AtCLC-g, were identified, completing the seven-member CLC family in *Arabidopsis* (AtCLC-a through AtCLC-g) [11]. These proteins display distinct subcellular localizations. For instance, AtCLC-a localizes to the vacuolar membrane, whereas AtCLC-e resides in the chloroplast thylakoid membrane, suggesting functional diversification among CLC family members.

Since the beginning of the 21st century, research has expanded from functional characterization of CLC proteins to the identification of additional chloride channels and chloride transport-related proteins. To date, several protein families responsible for Cl^−^ transport and anion movement have been identified in plants, such as ALMT, SLAC/SLAH, and NRT1/NPF proteins [8]. With advances in molecular biology, members of various Cl^−^ transport protein families have been cloned and characterized in model plants such as *Arabidopsis* [12] and rice (*Oryza sativa*) [13]. Moreover, the increasing availability of high-quality genome assemblies has accelerated the identification of chloride transport-related genes across diverse plant species. Consequently, functional research is gradually shifting from model plants to agriculturally important crops. Table 1 presents the reported number of genes belonging to major chloride channel and transporter families across representative plant species. As illustrated in Table 1, the reported gene copy numbers of the three major Cl^−^ transport-related families differ among species. In diploid species such as *Arabidopsis*, rice, maize, and soybean, the reported gene numbers of these families are relatively conserved. By contrast, polyploid species such as rapeseed, hexaploid wheat, and oat display gene family expansion, especially in the ALMT family, which contains 38 reported members in wheat. These observations indicate that genome ploidy and evolutionary polyploidization events may have contributed to the amplification of chloride transport-related genes, potentially increasing anion transport plasticity and environmental adaptation in higher plants.

## 3. Structural Characteristics of Gene Families Involved in Plant Chloride Transport and Homeostasis

Among the plant gene families involved in chloride transport and homeostasis, the CLC family has been the most extensively characterized, followed by the ALMT and SLAC/SLAH anion channel families. These proteins play pivotal roles in ion homeostasis, stress responses, and diverse physiological processes. Although they differ in domain composition, ion selectivity, transport mode, and subcellular localization, they collectively form a core molecular system that regulates plant Cl^−^ distribution, compartmentalization, and environmental adaptation.

### 3.1. CLC Gene Family

**Figure 1:**
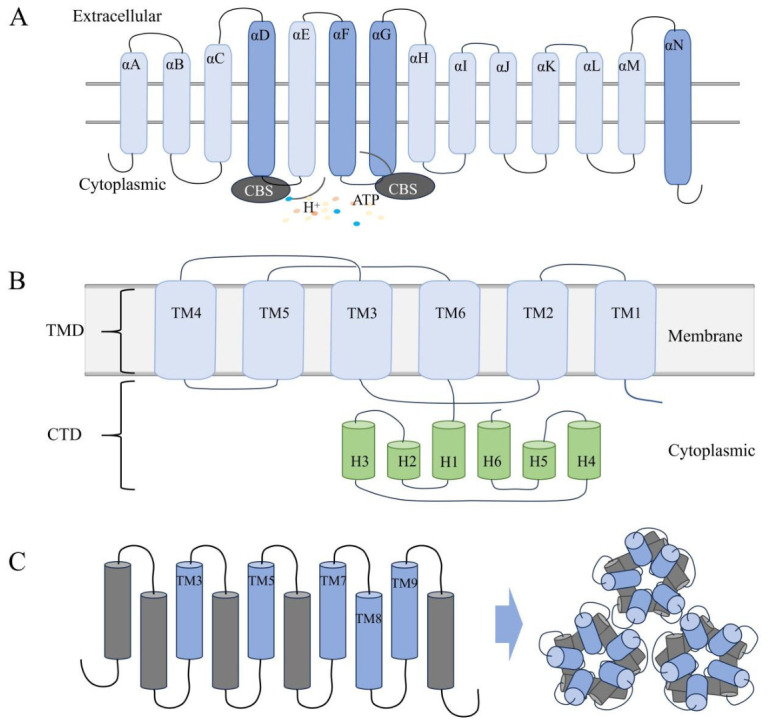
Typical structures of plant chloride ion-related protein families. (**A**) Two-dimensional structure of a CLC family monomer. (**B**) Two-dimensional structure of an ALMT family monomer. (**C**) Two-dimensional structure of an SLAC/SLAH family monomer. This figure was originally created by the authors using Adobe Illustrator 2020 (Adobe Inc., San Jose, CA, USA).

Plant CLC family proteins generally assemble as homodimers. Each subunit possesses an independent ion-conduction pathway and three Cl^−^ binding sites [20]. CLC family proteins are integral membrane proteins with 10–12 transmembrane domains [21]. Their pores contain two key functional sites: the selectivity filter (SF) and the proton glutamate (Glu_a_) residue, which is associated with gating and proton coupling. The SF is the core region responsible for Cl^−^-specific binding and selection. Eukaryotic CLC proteins generally contain two hydrophilic cystathionine β-synthase (CBS) domains, which modulate transport activity through binding with intracellular ATP, H^+^, and oxidized compounds [22] (Figure 1A). Plant CLC proteins contain three conserved amino acid motifs, GxGxPE, GKxGPxxH, and PxxGxLF, which are widely used as diagnostic signatures for CLC family identification. However, the degree of conservation of these motifs can vary among CLC subclasses and plant species [23,24]. Among these motifs, GxGxPE constitutes the selectivity filter [25,26], and the presence of serine within this domain determines Cl^−^ specificity [27,28].

In terms of subcellular localization, plant CLC members are mainly associated with the endomembrane system, including the vacuolar membranes (AtCLCa, AtCLCc, AtCLCg, GmCLC1, and ThCLC-a), Golgi apparatus (AtCLCd and AtCLCf), thylakoid membrane (AtCLCe), and mitochondria (ZmCLCc) [26]. These localization patterns are consistent with their roles in intracellular ion compartmentalization, organelle homeostasis, and function regulation of cellular physiology.

### 3.2. ALMT Family

ALMT family proteins primarily function as homodimers. Amino acid sequence analyses have shown that ALMT proteins contain multiple conserved domains, among which the aluminum-activated malate transporter domain (pfam11744) represents the defining structural component [29]. This domain can be divided into an N-terminal pore-forming transmembrane domain (TMD), which contains six transmembrane helices, and a C-terminal cytosolic domain (CTD) (Figure 1B). Except for ALMT14 isoforms 2 and 3, ALMT proteins generally contain a fusaric acid resistance protein-like domain (pfam13515), which extends from TM2 to TM6 and largely corresponds to the pore-forming region (Figure 1B). Within the transmembrane region, TM2 and TM5 form pore-lining helices at the channel center, while TM1, TM3, TM4, and TM6 are positioned at the periphery. The TMD contains an internal structural repeat unit in which TM1–3 and TM4–6 are related by a pseudo-two-fold axis parallel to the membrane. After TM6, helices H1–6 form an α-helical bundle that constitutes the CTD [30]. Phylogenetic analyses further divide closely related ALMT proteins into eight major clusters and several subclusters, suggesting structural and functional diversification within this family.

### 3.3. SLAC/SLAH Family

S-type anion channel homologs (SLAH) are widely expressed in plant tissues and play key roles in anion transport. SLAC/SLAH family proteins generally assemble as homotrimers. These membrane proteins contain 10 transmembrane helices (TMs), which in SLAC1 are arranged as helix-hairpin pairs to form a central five-helix transmembrane pore [31]. The transmembrane domains are designated TM1–TM10. Among them, TM3, TM5, TM7, TM8, and TM9 contribute to pore formation, while TM4, TM6, and TM10 help stabilize the trimeric structure. Extracellular inter-helix loops are short (2–5 residues), while intracellular inter-helix connections are longer and include a nine-residue helix (H2,3) between TM2 and TM3 (Figure 1C). Highly conserved positively charged residues on the cytoplasmic side may serve as potential interaction sites for regulatory kinases. Phosphorylation of serine/threonine residues can induce conformational changes in the pore-forming helices, thereby contributing to voltage-dependent gating [32]. The SLAC superfamily is divided into three subfamilies (SF1–SF3). SF1 comprises three large subfamilies, SF1A, SF1B, and SF1C, and plant SLAC/SLAH proteins belong to the SF1A subfamily [31]. In *A. thaliana*, this family comprises five members: SLAC1 and four homologs, SLAH1–SLAH4. SLAC1 is predominantly expressed in guard cells and serves as a key regulator of stomatal closure. Among the SLAH members, SLAH3 shows overlapping expression with SLAC1 in guard cells but is also expressed in roots, where it facilitates the long-distance NO_3_^−^ and Cl^−^ transport from roots to shoots. SLAH2 is mainly expressed in root stele cells and plays a critical role in nitrate acquisition and transport within the vascular system [33,34,35,36].

### 3.4. CCC, NPF, and MATE Families in Relation to Chloride Balance

Beyond the major chloride-conducting families CLC, ALMT, and SLAC/SLAH, the CCC (cation-chloride cotransporter), NPF (nitrate/peptide transporter), and MATE (multidrug and toxic compound extrusion) families are also closely linked to plant Cl^−^ balance, either through direct transport or indirect regulation. Plant CCC proteins are long polypeptides of approximately 700–1200 amino acids and form two distinct clades (CCC1 and CCC2). Compared with CCC2 family proteins, CCC1 proteins have a shorter amino N-terminus and an insertion near the C-terminus [37]. These structural differences may be associated with functional diversification in potassium/sodium ion (K^+^/Na^+^)-coupled Cl^−^ cotransport. The NPF family is one of the largest transporter families in plants. Most NPF members function as proton-coupled symporters, and proton coupling appears dependent on a conserved ExxER/K motif.

NPF proteins are commonly divided into NRT1 (low-affinity nitrate transporters) and PTR (oligopeptide transporters) categories. These proteins include members involved in nitrate, peptide, hormone, and other substrate transport; however, their contribution to Cl^−^ transport is member-specific and should not be generalized to the entire family. Although Cl^−^ is not the primary substrate of most NPF proteins, some NPF members can influence Cl^−^ homeostasis directly or indirectly because nitrate (NO_3_^−^) and Cl^−^ may share or compete for anion transport pathways. Thus, specific NPF members may indirectly modulate root Cl^−^ acquisition by altering the balance between NO_3_^−^ and Cl^−^ transport, although this function should be interpreted on a gene-specific basis [38,39]. The MATE family may also indirectly affect Cl^−^ homeostasis. MATE monomers contain 12 transmembrane α-helices (TM1-TM12) and predominantly function as homodimers [40]. By extruding organic acids and secondary metabolites, MATEs may alter cytosolic pH and electrochemical gradients under stress conditions. Collectively, these families are functionally diverse but closely linked to the ability of plants to maintain Cl^−^ balance under fluctuating environmental conditions.

## 4. Core Physiological Functions of Plant Chloride Transport Proteins

### 4.1. Cl^−^ Uptake and Maintenance of Intracellular Ion Homeostasis

Plant chloride transport proteins participate in Cl^−^ uptake, long-distance transport, and intracellular distribution. Plants absorb Cl^−^ from soil through the roots and then distribute it to roots, stems, and leaves via the synergistic action of chloride transport proteins to promote growth. Specific NPF family members may contribute to Cl^−^ uptake or Cl^−^ homeostasis, but this function should be assigned only when supported by direct transport or mutant evidence. Root Cl^−^ uptake may occur through apoplastic, symplastic, or transcellular pathways [41]. After entering the epidermis, Cl^−^ can move between cortical cells through plasmodesmata via the symplastic pathway. When Cl^−^ reaches the endodermis, the Casparian strip restricts apoplastic movement and forces ions to cross the plasma membrane into the endodermal cytoplasm, thereby enabling transcellular transport. Activation of anion channels can depolarize the plasma membrane of xylem parenchyma cells and promote Cl^−^ and K^+^ extrusion into xylem vessels. Slow (S)-type anion channels have been reported to be active in xylem parenchyma cells [42]. In *A. thaliana*, the SLAH3 channel interacts with the non-functional SLAH1-subunit, which enhances SLAH3 activity and Cl^−^ conductance. This finding indicates that SLAH3/SLAH1 heteromeric channels are important for long-distance Cl^−^ transport through the xylem [43].

Plant chloride transport proteins maintain intracellular Cl^−^ homeostasis primarily through sequestration and efflux. In *Arabidopsis*, all CLC proteins identified to date localize to intracellular compartments, and plasma membrane-localized CLC-type channels have not yet been confirmed [44]. Excess Cl^−^ can be sequestered in vacuoles through the activity of vacuolar membrane-localized chloride transport proteins, thereby reducing cytosolic Cl^−^ toxicity. For instance, under salt stress, AtCLCf translocates from the Golgi apparatus to the plasma membrane in *Arabidopsis* roots, where it contributes to Cl^−^ efflux and removes excess Cl^−^ from roots, thereby enhancing salinity tolerance [45]. This “uptake-transport-compartmentalization” model is crucial for maintaining Cl^−^ homeostasis under fluctuating environmental Cl^−^ concentrations.

### 4.2. Involvement in Plant Abiotic Stress Responses

Plant chloride transport proteins contribute to adaptation under environmental stressors, such as low temperatures and salinity, by regulating ion balance, osmotic adjustment, and cellular physiological status. Under low-temperature stress, activation of plant chloride channels can mediate Cl^−^ efflux, contributing to maintaining osmotic homeostasis, reducing cold-induced cellular dehydration, protecting membrane integrity, and improving cold tolerance. *ZmCLC-c* has been reported to play an essential role in cold resistance in maize. *ZmCLC* gene expression was induced in cold-stressed maize seedlings, and *ZmCLC-c* expression was significantly higher in cold-tolerant varieties during seed germination and early seedling growth [46].

Under high-salinity conditions, chloride transport proteins help maintain intracellular ion homeostasis, osmotic balance, and normal metabolism by regulating Cl^−^ transmembrane transport and compartmentalization. Heterologous expression of *CsCLC-c* in *Arabidopsis* improved seed germination under salt stress. Additionally, total Cl^−^ accumulation in the roots and stems of transgenic plants was lower than that in mutants or wild-type plants, indicating that *CsCLC-c* plays a key role in Cl^−^ homeostasis and may represent a potential target for improving plant salt tolerance [47]. Overexpression of *GsCLC-c2* contributed to Cl^−^ and nitrate homeostasis by increasing Cl^−^ accumulation in roots and reducing its transport to tissues that are more prone to salt-induced damage [18]. In the halophyte *Suaeda glauca*, the expression levels of *SaCLC-d*, *SaCLC-f*, and *SaCLC-g* increased in leaves under different salt concentrations. This response was accompanied by intracellular Cl^−^ aggregation, suggesting that these proteins may participate in salt concentration-dependent Cl^−^ sequestration [27]. In cotton seedlings, silencing of *GhCLCc-1* gene increased Cl^−^ accumulation in roots, stems, and leaves under salt treatment, resulting in reduced salt tolerance. Conversely, ectopic expression of *GhCLCc-1* gene in *Arabidopsis* reduced Cl^−^ accumulation in transgenic lines under salt stress and enhanced salt tolerance [48].

### 4.3. Regulation of Stomatal Movement

Stomata are formed by pairs of specialized guard cells in leaves and serve as gateways for transpiration and CO_2_ influx during photosynthesis [49,50]. Precise regulation of stomatal movement is critical for plant adaptation to changing environmental conditions, and chloride channels and anion transport systems play central roles in this process. In *Arabidopsis*, *SLAC/SLAH* genes encode S-type anion channels that are key regulators of stomatal dynamics [51,52]. In response to abscisic acid (ABA), these channels are activated and mediate Cl^−^ efflux from guard cells, causing plasma membrane depolarization [53]. This depolarization subsequently activates K^+^ channels, promotes K^+^ release, reduces guard cell turgor, and ultimately induces water loss and stomatal closure [54]. Modulation of *SLAC*1 expression can substantially affect stomatal conductance. For instance, *Arabidopsis* lines overexpressing SLAC1 exhibit accelerated stomatal closure under drought conditions. In a recent study, *DcaSLAC*1, a candidate gene involved in stomatal regulation, was identified from the traditional orchid *Dendrobium officinale* through phylogenetic and expression analyses. Heterologous expression of *DcaSLAC1* in wild-type *Arabidopsis* and the *slac1* mutant enhanced drought tolerance in transgenic plants by reducing stomatal conductance [55].

ALMT family proteins, which are plant-specific anion channels, are also involved in stomatal regulation. In *Arabidopsis*, loss of aluminum-activated malate transporter 12 (ALMT12) stimulates stomatal opening and inhibits multiple stomatal closure responses, suggesting that ALMT12 contributes to R-type anion channels activity [56,57]. SLAC1 and R-type ALMT12 function as independent anion-permeable channels that mediate anion efflux from guard cells during stomatal closure. However, they may also interact with each other and modulate each other’s activity [58].

### 4.4. Regulation of Other Growth and Development Processes

Chloride transport proteins participate in multiple stages of plant growth and development, from seed germination to reproductive growth. Virus-induced silencing of the tomato (*Solanum lycopersicum* L.) chloride channel gene *SlCLC-b* causes stunted growth and leaf curling, indicating the important role of *SlCLC-b* in plant development [59]. Vacuolar membrane-localized CLC-type proteins regulate vacuolar Cl^−^ accumulation and release, affecting vacuolar osmotic potential, thereby regulating cell turgor, cell expansion and elongation, radicle protrusion, and successful seed germination [28]. In oat, MSN*-siAsCLC11/13/25* complexes were prepared by co-incubating siRNA with mesoporous silica nanoparticles (MSN) and then applied to leaves via foliar spraying. Under salt stress, plants with silenced *AsCLC11*, *AsCLC13*, and *AsCLC25* exhibited increased salt sensitivity and reduced photosynthetic performance.

Genes silencing also impaired the antioxidant defense system, indicating that *AsCLC11*, *AsCLC13*, and *AsCLC25* are important for maintaining photosynthetic performance and oxidative stress resistance under saline-alkaline conditions [19]. In *Arabidopsis*, *AtALMT12* regulates stomatal closure and mediates the transmembrane transport of Cl^−^ and other anions. By maintaining stomatal movement and water balance, *AtALMT12* may also contribute to reproductive development and help prevent flower wilting under stress [57].

**Figure 2:**
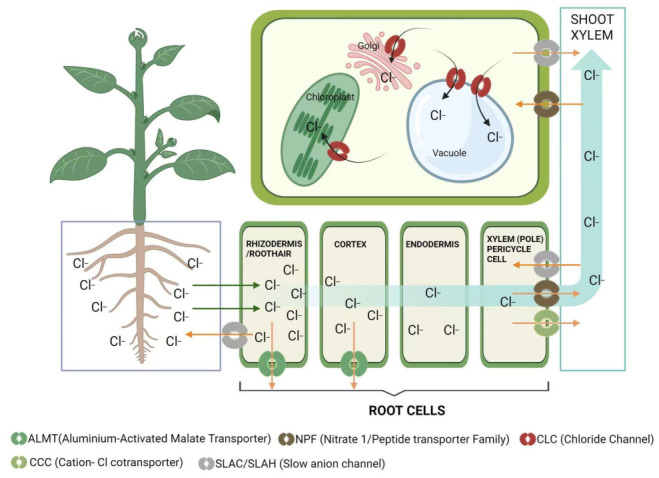
Schematic diagram illustrating the whole-plant chloride transport pathway mediated by different chloride channel and transporter proteins. Cl^−^ uptake from soil by root epidermal cells, radial transport across cortex and endodermis, xylem loading for long-distance translocation to shoots, and intracellular Cl^−^ compartmentation into vacuoles, chloroplasts and Golgi apparatus in leaf cells are displayed. Five types of key Cl^−^ transport proteins (CLC, SLAC/SLAH, NPF, CCC, ALMT) are marked at their corresponding functional sites. This figure was originally created with BioRender.com and retrieved from https://BioRender.com/6t74pd5, accessed 7 July 2026.

Collectively, multiple chloride transporters coordinate whole-plant Cl^−^ absorption, long-distance translocation, and intracellular compartmentalization to maintain ion homeostasis. The integrated transport pathways mediated by CLC, SLAC/SLAH, NPF, CCC and ALMT families are summarized in Figure 2.

## 5. Regulatory Mechanisms of Chloride Transport Proteins

### 5.1. Phosphorylation and Dephosphorylation Regulation

**Figure 3:**
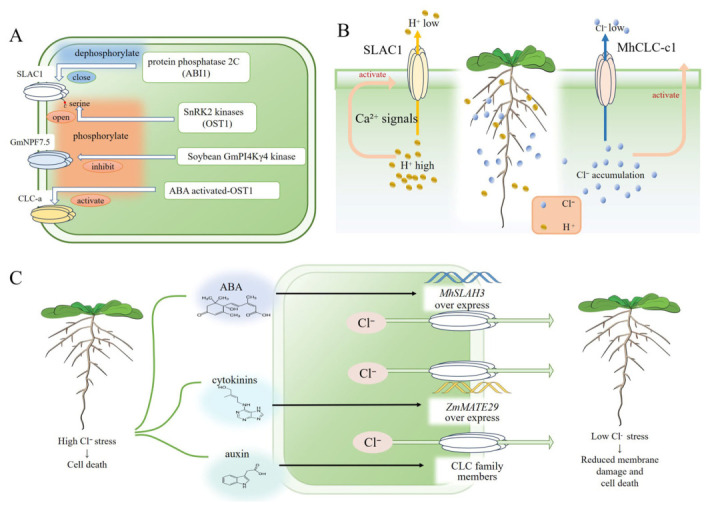
Schematic diagram of the regulatory mechanisms of plant chloride transport proteins in response to chloride stress. (**A**) Phosphorylation–dephosphorylation loop. (**B**) Ion and pH regulation. (**C**) Phytohormone signaling modulation. This figure was originally created by the authors using Adobe Illustrator 2020 (Adobe Inc., San Jose, CA, USA).

Phosphorylation is one of the major post-translational mechanisms regulating the activity of chloride channel proteins. In *Arabidopsis*, phosphorylation of SLAC1 precisely regulates the balance between basal and activated trimer states, thereby controlling stomatal aperture. In the abscisic acid (ABA) signaling pathway, SLAC1 activity is regulated by the kinase-phosphatase pair (OST1/ABI1). Following ABA signal activation, SnRK2 kinases, including OST1, are activated by phosphorylation. These kinases phosphorylate serine residues in the N-terminus of SLAC1 and promote the transition of SLAC1 channel from a closed to an open state. Conversely, protein phosphatase 2C members, including ABI1, inhibit SLAC1 activity through dephosphorylation, forming a “phosphorylation-dephosphorylation” regulatory loop [60,61,62] (Figure 3A).

Fourteen phosphorylation sites have been identified in AtSLAC1, and phosphorylation of 4–6 of these sites increases channel activity by nearly 330-fold [63]. CLC family members are also regulated by phosphorylation. The ABA-activated kinase OST1 phosphorylates the Thr38 site in the N-terminal domain of AtCLC-a, increasing outward anion flux across the vacuolar membrane and enhancing anion release from vacuoles. This finding reveals that phosphorylation-mediated regulation is involved in the control of CLC activity in eukaryotic cells [64]. The activity of GmNPF7.5 is similarly regulated by phosphorylation. Using yeast two-hybrid technology, GmPI4Kγ4 was identified as an interacting partner of GmNPF7.5. This soybean kinase specifically inhibited the Cl^−^ transport activity of GmNPF7.5 through phosphorylation without affecting its NO_3_^−^ transport efficiency. This post-translational modification reduced Cl^−^ accumulation and enhanced salt tolerance in soybean. Therefore, modulation of GmNPF7.5 activity provides a mechanism through which plants can more precisely regulate intracellular Cl^−^ levels and improve adaptation to saline-alkaline environments [8].

### 5.2. Regulation by Ion Concentration and pH

Intracellular ion concentrations and cytoplasmic pH are important regulators of chloride transport protein activity. Among these proteins, CLC members are highly associated with Cl^−^ accumulation and plant tolerance to sodium chloride (NaCl) stress [65]. Expression analysis of *MdCLC* homologs in *Malus hupehensis* roots showed that most *MhCLC* genes respond to NaCl stress. In particular, *MhCLC-c*1 was rapidly and continuously upregulated under NaCl treatment. Functional analysis indicated that *MhCLC-c*1 inhibited intracellular Cl^−^ accumulation under NaCl stress, thereby alleviating NaCl-induced cell death [66] (Figure 3B).

The opening of SLAC1 channels is also sensitive to cytoplasmic pH. A decrease in cytoplasmic pH can enhance SLAC1 activity, which may help maintain normal channel function under acidic stress. In this process, a hydrogen ion (H^+^) can induce cytoplasmic calcium ion (Ca^2+^) signaling and promote membrane depolarization by activating Ca^2+^-dependent SLAC1/SLAH3 anion channels, thereby contributing to long-distance regulation of stomatal movement [67] (Figure 3B). A systems dynamics model of *Arabidopsis* further explained the paradoxical phenotype caused by SLAC1 anion channel mutations. The model suggested that anion accumulation in mutants inhibits cytoplasmic H^+^ loading and promotes Ca^2+^ influx, leading to increased cytoplasmic pH (pHi) and elevated free cytoplasmic Ca^2+^ concentration ([Ca^2+^]i) [68].

### 5.3. Regulation by Hormone Signaling

Plant hormones regulate the expression and activity of chloride transport proteins through downstream signaling pathways (Figure 3C). ABA is a key regulator of stomatal movement, and its signaling pathway is closely associated with the activity of anion channel proteins such as SLAC proteins and some CLC members. In *Malus hupehensis*, exogenous ABA application improved Cl^−^ efflux from roots and alleviated membrane damage and cell death under Cl^−^ stress. *MhSLAH3* was co-induced by Cl^−^ and ABA, and its overexpression accelerated Cl^−^ efflux, thereby enhancing tolerance to Cl^−^ stress and delaying Cl^−^-induced cell death [69].

In addition to ABA, cytokinins also play important regulatory roles in plant stress responses. In maize, cytokinin signaling enhances Cl^−^ exclusion, with the vacuolar membrane-localized chloride transporter *ZmMATE29* acting as a core regulatory target. Upregulation of *ZmMATE29* promoted cytoplasmic Cl^−^ compartmentalization into vacuoles, reducing Cl^−^ accumulation and enhancing salt tolerance [70]. Although direct evidence that auxin binds to CLC-type channel proteins is lacking, recent studies suggest that auxin may directly or indirectly regulate the expression of CLC family members, thereby influencing Cl^−^ transport during root growth and stress responses [71]. Overall, the synergistic effect of hormone signals and chloride transport proteins enables plants to adjust Cl^−^ transport according to growth requirements and environmental conditions.

## 6. Research Technologies and Application Prospects

### 6.1. Advances in Technologies for Chloride Transport Gene Mining and Application

Research on plant chloride transport proteins has advanced rapidly with the development of molecular, physiological, and structural biology technologies. Homologous sequence alignment and conserved domain analysis have enabled researchers to predict and clone multiple chloride channel genes from various plants [10]. For gene cloning and functional verification, CRISPR/Cas9-mediated genome editing has been widely used to generate mutants of candidate channel and transporter genes [72]. When combined with genetic transformation systems in model plants such as *Arabidopsis* and rice, this technology allows rapid assessment of gene function. In addition, the increasing availability of high-quality plant genome assemblies has made bioinformatics an essential tool for genome-wide identification of chloride channel and transport-related gene families.

Cryo-Electron Microscopy (Cryo-EM) has become a core technique for resolving high-resolution three-dimensional structures of biological macromolecules. In plant chloride channel research, Cryo-EM has provided direct structural evidence on channel spatial conformation, ion selectivity mechanisms, and gating regulation [30]. Electrophysiological techniques, particularly the patch-clamp technique, remain classic and direct methods for characterizing plant chloride channels. These techniques allow researchers to detect current signals generated by Cl^−^ transmembrane flow, analyze voltage-dependent gating characteristics, and examine the effects of hormones and ion concentrations on channel activity [73].

### 6.2. Application Prospects of Chloride Channel and Transporter Genes in Agricultural Production

Functional studies suggest that chloride channel and transporter genes hold potential applications in crop improvement. In stress-tolerant crop breeding, genome editing and transgenic approaches can be used to modify genes involved in plant stomatal regulation, ion homeostasis, and drought and salt tolerance. For example, chloride-related channels participate in stomatal opening and closure, thereby affecting transpiration and water-use efficiency. Modulation of *SLAC1* expression may therefore contribute to the cultivation of crop varieties with reduced stomatal conductance and improved water use efficiency, particularly in arid agricultural regions [55]. The CLC family also exhibits considerable potential for improving salt-alkaline tolerance, although this potential requires further validation under crop-specific and field conditions. Future studies may use transgenic or genome editing technologies to modify CLC genes in staple crops such as wheat and maize, with the aim of developing varieties with improved tolerance to saline-alkaline lands. However, these applications require careful evaluations under field conditions before large-scale agricultural use. In terms of nutrient utilization, Cl^−^ is a key cofactor in the chloroplast thylakoid electron transport chain and contributes to photosystem II stability [74]. Therefore, optimizing chloride transport may contribute to improved photosynthetic efficiency and biomass accumulation, depending on species, developmental stage, and environmental conditions. For example, manipulating chloride transporter activity in crops such as maize and soybean may contribute to short growth cycle and increase grain yield [75]. The synergistic transport of Cl^−^ with cations such as K^+^ and Na^+^ is crucial for nutrient uptake. Genetic strategies that balance Cl^−^ to K^+^ homeostasis may improve trace element utilization, reduce fertilizer waste, and lower production costs.

Despite these promising prospects, the application of chloride channel and transporter genes may also have negative impacts on plant growth and development. In *Arabidopsis*, *AtCLCd* acts as a negative regulator of pathogen-associated molecular pattern (PAMP)-triggered immunity (PTI) [76]. Excessive expression of chloride transporters may disrupt intracellular Cl^−^ homeostasis and cause Cl^−^ over-accumulation and phytotoxicity. Suppression of *GmNPF7.5^HapA^* expression decreases Cl^−^ accumulation and salt damage, whereas its overexpression produces the opposite effect [8]. Overexpression of *GmNPF6.8* in *Arabidopsis* and soybean hairy roots significantly reduced root length and root density. It also altered the expression of key genes involved in root development [77]. These findings indicate that future translational research must combine precise gene expression control with multi-location field trials to evaluate agronomic performance, nutrient-use efficiency, and environmental safety. Such approaches are necessary to enhance stress tolerance and productivity without compromising crop fitness.

## 7. Research Outlooks and Challenges

Research on plant chloride channels and transport proteins has substantially advanced our understanding of plant anion transmembrane transport. These studies have clarified interactions between Cl^−^ and other ions, such as K^+^ and H^+^, and have revealed the molecular chain reaction of “ion balance-cell function-plant growth”. This knowledge has enriched the theoretical framework of plant mineral nutrition physiology. Chloride channels and transporters also contribute to plant responses to abiotic stress. Studies of their functions and regulatory mechanisms can clarify how plants perceive stress signals, activate channel activity, and initiate stress resistance responses, thereby deepening our understanding of plant environmental adaptability.

Plant chloride transport families (e.g., CLC, SLAC/SLAH, ALMT) play core roles in crop ion homeostasis, abiotic stress responses, stomatal movement, and nutrient metabolism. Targeted manipulation of these proteins through gene overexpression, gene silencing, or genome editing may provide strategies for improving crop salt tolerance and yield stability. For example, the upregulated expression of two slow anion channel genes (*HvSLAH1* and *HvSLAC1*) in barley is closely associated with salt tolerance and grain yield [78]. Overexpression of these genes enhanced stomatal regulation under salt stress, reduced water loss, helped maintain photosynthetic efficiency, and was associated with increased grain yield.

Despite significant progress in current research on plant chloride channel and transporter genes, numerous challenges remain. The complexity of plant cell membranes makes the purification and in vitro reconstitution of chloride channels and transporters extremely difficult. In addition, plant chloride transport families have numerous members with partially overlapping functions, which complicates the interpretation of single-gene knockout phenotypes. Additionally, translating basic research into field applications remains challenging because environmental regulation of channel activity and chloride transport is not yet fully elucidated. Although the salt tolerance potential of specific channel genes has been verified under controlled laboratory conditions, their effects may vary in field trials due to soil heterogeneity, climate fluctuations, and interactions with other stress factors. Public acceptance of transgenic crops and the need for ecological risk assessment further complicate technology implementation.

Future research should focus on the functional mechanisms of chloride transport proteins, including the interactions among proteins localized in different organelles and their cross-talk with other ion channels, such as potassium and calcium channels. Understanding these interactions will be critical for clarifying plant ion balance regulation. Further exploration of chloride channel and transporter genes is also needed for crop improvement. Candidate genes may be modified or introduced through gene editing, genetic transformation, or molecular breeding to breed crop varieties with enhanced tolerance to salinity, drought, low temperatures, and other stresses. These strategies may improve crop yield and quality under harsh environmental conditions and contribute to food security. The potential roles of chloride channels and transporters in plant nutrient utilization efficiency, such as nitrogen and phosphorus utilization, should also be investigated. Such studies may provide theoretical basis and genetic resources for breeding high-yield and resource-efficient crop varieties, reducing fertilizer use, lowering production costs, and mitigating environmental pollution. Future research should also strengthen technological innovation and interdisciplinary integration. Systems biology approaches combined with multi-omics technologies (e.g., transcriptomics, proteomics, metabolomics) can be used to comprehensively analyze the regulatory networks and signaling pathways associated with chloride channels in plant cells. In summary, future research should integrate mechanistic studies, technological innovation, and translational applications to advance the role of chloride transport biology in agriculture and environmental sustainability.

## Figures and Tables

**Table 1:** Reported number of genes in several chloride channels and transporters across representative plant species.

No.	Species	Latin Name	Chromosome Ploidy	Number of Gene Family Members			References
				CLC Family	ALMT Family	SLAC/SLAH Family	
1	*Arabidopsis*	*Arabidopsis thaliana* L.	Diploid	7	14	5	[12]
2	Rice	*Oryza sativa* L.	Diploid	6	10	4	[13]
3	Maize	*Zea mays* L.	Diploid	5	12	4	[14,15]
4	Rapeseed	*Brassica napus* L.	Allotetraploid	7	23	4	[16]
5	Wheat	*Triticum aestivum* L.	Hexaploid	12	38	5	[17]
6	Soybean	*Glycine max* (L.) Merr.	Diploid	10	18	5	[18]
7	Oat	*Avena sativa* L.	Hexaploid	8	11	3	[19]

## Data Availability

All data supporting the findings of this study are provided in the main manuscript.
